# Characterization of the spatial distribution of plateau pika burrows along an alpine grassland degradation gradient on the Qinghai–Tibet Plateau

**DOI:** 10.1002/ece3.8176

**Published:** 2021-10-07

**Authors:** Dawen Qian, Qian Li, Bo Fan, Yuting Lan, Guangmin Cao

**Affiliations:** ^1^ Key Laboratory of Cold Regions Restoration Ecology Qinghai Province Northwest Institute of Plateau Biology Chinese Academy of Sciences Xining China

**Keywords:** alpine grassland, landscape pattern, pika burrow, Qinghai–Tibet Plateau, UAV

## Abstract

Plateau pika burrows are common feature of degraded grassland in the Qinghai–Tibet Plateau (QTP) and serve as an important indicator of pika activity and grassland degradation. However, the current understanding of the spatial pattern changes of pika burrows and their critical thresholds across a degradation gradient in alpine grassland is deficient. In this study, we investigated and quantified changes in the spatial pattern of plateau pika burrows under typical degraded alpine shrub meadows in the northeastern QTP using an unmanned aerial vehicle and landscape pattern metrics. The degradation of the alpine shrub meadow leads to a change in landscape pattern from a two‐layered structure of alpine shrub and alpine meadow to a mosaic of alpine meadow and bare soil, with plateau pika burrows scattered throughout. Moderate degradation is the tipping point for changes in surface landscape patterns, followed by the disappearance of alpine shrub, the retreat of alpine meadows and the encroachment of bare soil, and the increasing density and size of pika burrows. The area characteristics of alpine meadows have influenced changes in the spatial pattern of pika burrow, and maintaining its proportional area is a vital measure to control the threat of pika burrows to pastures. The results of this paper provide a methodological reference and guidance for the sustainable utilization of grassland on the QTP.

## Graphical abstract

UAV‐based spatial pattern survey of plateau pika burrows. Negative effect of alpine meadow area ratio on pika burrow density. Spatial pattern characteristics of pika burrows will support grassland management.

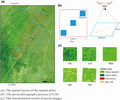

## 1 INTRODUCTION

The Qinghai–Tibet Plateau, known as the “roof of the world” due to its high altitude, has a unique climate and ecosystem that are profoundly affected by climate change and impact on water resources and biodiversity conservation in the region and beyond. The alpine grasslands cover 60% of the plateau and are the important material source for the livelihoods of 5 million pastoralists (Dong & Sherman, 2015; Harris, 2010). Alpine grassland also plays an important role in water conservation, hydrological regulation, and biodiversity conservation on the plateau (Liu et al., 2018; Niu, Zhou, et al., 2019; Wu et al., 2017). Nearly half of the alpine grassland has been experiencing degradation for several decades on QTP (Dong & Sherman, 2015), resulting in decreased plant diversity and productivity, accelerated soil erosion (Liu et al., 2018), and an increase in greenhouse gases released into the atmosphere (Su et al., 2015). There are many potential causes of alpine grassland degradation, and it is almost certain that the degradation results from a combination of many factors, including overgrazing, climate change, frozen soil, and soil disturbance from small mammals (Cao et al., 2019; Harris, 2010).

The plateau pika (*Ochotona curzoniae*) has long been considered to be responsible for the degradation of alpine grassland due to their foraging, which can reduce vegetation height and alter plant community structure and biomass, and due to their burrowing behavior, which can destroy surface vegetation and increase the risk of soil erosion (Yi et al., 2016; Yu et al., 2017; Zhang et al., 2020). However, as research into the mechanisms behind alpine grassland degradation has increased, there is a growing consensus that the plateau pika is more of a consequence than a cause of grassland degradation. Plateau pikas provide many benefits for grassland ecosystems; for example, their burrows can provide shelter for other animals and their behavior serves to increase vegetation species richness, and they are also the prime prey for predator species on QTP (Dobson et al., 2019; Smith et al., 2019).

The burrows are important places for plateau pika to shelter, rest, and breed. Pika burrows can have detrimental or beneficial effects on grassland ecosystems (Sun et al., 2015); they not only can reduce plant productivity, soil organic carbon, and total nitrogen and can increase greenhouse gas emissions (Qin, Yi, et al., 2020; Zhao et al., 2019), but they can also provide homes for other animals and increase soil water infiltration efficiency (Delibes‐Mateos et al., 2011; Wilson & Smith, 2015). In addition, the presence and development of pika burrows can strongly alter the spatial pattern of grassland (Tang et al., 2019). The fragmentation of intact grassland patches through the direct destruction and occupation of pika burrows, combined with the continued degradation of grassland and trampling by livestock, leads to the further expansion of bare patches (Cao et al., 2019; Chen et al., 2017; Niu, Zhu, et al., 2019). Therefore, pika burrows are considered by pastoralists as a sign of grassland degradation and an obstacle to the sustainable use of rangelands. This is the main reason for the long‐standing conflict between herders and plateau pikas, thus prompting herders to frequently use trapping and poisoning to exterminate the pikas (Pang et al., 2020).

The spatial pattern of pika burrows can provide us with information on the population size and activity patterns of plateau pikas, as well as serve as a basis for determining the degree of grassland degradation (Wei et al., 2019, 2020; Zhao et al., 2019). The traditional survey method is manual field measurements of the burrows, but it still has the shortcomings of being time‐consuming and costly. The rapid development of unmanned aerial vehicle (UAV) in recent years has opened new methods for ecological research. UAVs have a fast revisit time and can provide data at high spatial resolution and low cost, which overcome the shortcomings of traditional surveys (Anderson & Gaston, 2013; Manfreda et al., 2018). A variety of studies have been conducted using UAV technology to investigate the spatial patterns of plateau pika burrows and their effects on surrounding grassland cover and bare patches (Tang et al., 2019; Zhang et al., 2021), as well as on carbon emissions, ecosystem respiration, vegetation biomass, and soil organic carbon (Qin et al., 2015, 2019; Qin, Yi, et al., 2020). However, these studies have only focused on the spatial pattern of plateau pika burrows under a single level of degradation, and lack an understanding of the spatial pattern changes of plateau pika burrows and other types under the complete degradation gradient of alpine grasslands, so the key thresholds for the changes of pika burrow patterns are also unclear.

We investigated an alpine grassland pasture with a complete degradation sequence in the northeastern Qinghai–Tibet Plateau using an unmanned aerial vehicle. The objective of this paper was to quantitatively assess the changes in spatial pattern characteristics of plateau pika burrows and other surface types under different degrees of degradation in alpine grasslands, as well as the quantitative relationships between them, and thus obtain information on the key thresholds of pika burrow patterns corresponding to different degrees of grassland degradation. The results of this paper will improve our understanding of the changing pattern of pika burrows in degraded grassland on the QTP and provide a valuable reference for grassland management and wildlife conservation on the QTP.

## 2 MATERIALS AND METHODS

### 2.1 Study site and field sampling

#### 2.1.1 Study site

**FIGURE 1:**
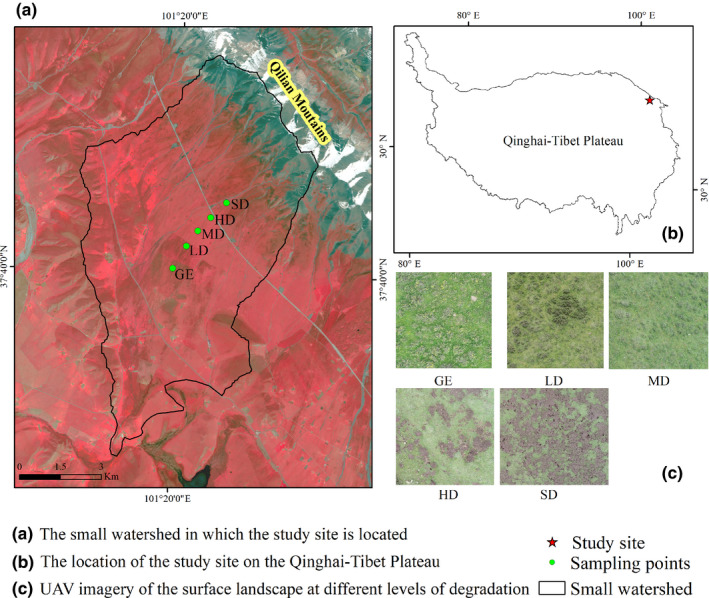
Study site location. Note: Figure a, b, and c is the small watershed in which the study site is located, the location of the study site on the Qinghai–Tibet Plateau, and UAV imagery of the surface landscape at different levels of degradation, respectively. Abbreviations: GE, grazing exclusion; HD, heavy degradation; LD, light degradation; MD, moderate degradation; SD, severe degradation

The study site is located on the northeastern edge of the Qinghai–Tibet Plateau, in an area of premountain pasture formed by alluvial fans on the southern slope of the Qilian Mountains, and is part of Menyuan County, Qinghai Province, China (Figure 1). The region has a typical continental plateau climate with only two seasons a year: a cold and dry winter, and a humid and rainy summer. The average annual temperature is −1.7°C, with the lowest temperature occurring in January, when it can reach −14.8°C, and the highest temperature occurring in July, when it can reach 9.8°C. The total annual precipitation is 580 mm and is concentrated between May and September, which accounts for 80% of the total annual precipitation (Cao et al., 2007).

The vegetation type is alpine shrub meadow, with a two‐layer structure that comprises an upper shrub layer (*Potentilla fruticosa* as the dominant species) and a lower herbaceous layer (dominated by *Kobresia humilis*). The area is used by local herders as summer pasture, it is grazed from June to September by Tibetan sheep and yaks. The short grazing period and the mixture of different grazing management regimes (family, joint family, and communal pastures) lead to more intensive grazing. Such grazing management has been practiced for at least 30 years, this has resulted in the degradation of previously widespread alpine shrub meadows from north to south, as is evidenced by a decline in the canopy, height, and proportion of shrub, and changes in the structure of plant communities and landscape patterns (Dai et al., 2020).

#### 2.1.2 Field sampling

Along the degradation gradient, we used the coverage of potentilla *fruticosa shrub*, the number of plant species, and the aboveground biomass to classify the degree of degradation into light degradation (LD), moderate degradation (MD), heavy degradation (HD), and severe degradation (SD), and to choose a grazing exclusion (GE) site as nondegraded alpine grassland (enclosed for more than 4 years) (Table 1) (Dai et al., 2020).

**TABLE 1:** Classification of alpine grassland degradation levels

Degradation level	*Potentilla fruticosa* shrub coverage (%)	Species number	Aboveground biomass (g/m^2^)
GE	50–60	24	396
LD	40–50	22	347
MD	5–10	26	283
HD	0	16	202
SD	0	14	189

**FIGURE 2:**
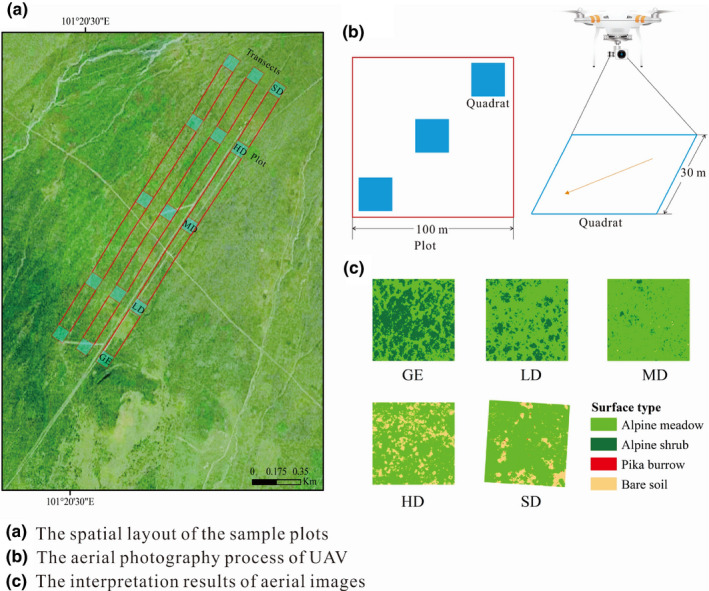
Field sampling and UAV aerial photography. Note: a, b, and c is the spatial layout of the sample plots, the aerial photography process of UAV, and the interpretation results of aerial images. Abbreviations: GE, grazing exclusion; HD, heavy degradation; LD, light degradation; MD, moderate degradation; SD, severe degradation

We arranged three plots (100 m × 100 m) in each degraded sample site at an average interval of approximately 200 m. In each plot, three quadrats (30 m × 30 m) were placed and photographed by the UAV along the diagonal within each sample plot, which were equidistantly distributed at an approximate interval of 30 m. A total of 15 plots and 45 quadrats were laid out (Figure 2).

For each plot, a UAV (Phantom 4 Pro; DJI Innovation Company), controlled by DJI GS PRO software, was used to take photographs of the quadrats with the camera looking down vertically. The Phantom 4 Pro has a camera with a 1/2.3″ CMOS sensor and 20 million pixels, the lens is 24 mm (35 mm format equivalent) with a wide (84°) field of view angle. We chose clear weather and light winds for our aerial photography work, which was carried out at around 12 p.m. on 28 June and 5 July 2018. The flying height is 30 m, and the ground area covered by each photograph is approximately 37 m × 30 m, with a ground resolution of 1.5 cm per pixel.

### 2.2 Data processing

#### 2.2.1 UAV data processing and surface type classification

**FIGURE 3:**
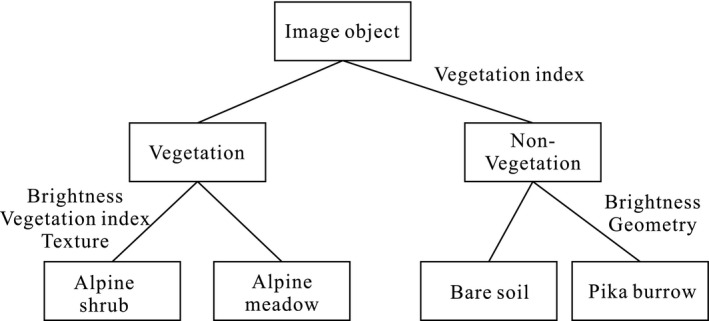
Flowchart of decision tree classification

The UAV imagery in each quadrat was processed using Agisoft PhotoScan to produce the remote‐sensing images. We classified the surface types into alpine shrub, alpine meadow, bare soil, and plateau pika burrow using an object‐oriented classification technique with the eCognition 9.0 software, consisting of multiresolution segmentation and decision tree classification. The multiresolution segmentation had a segmentation scale of 15, and a shape and compactness parameter of 0.5, which were obtained by trial and error. A simple decision classification tree was then constructed (Figure 3), and the images were classified by combining key indicators of the image objects, that is, the spectral, spatial, and textural information that was representative of the object (Table 2). The classification results were then validated to assess the accuracy, and the decision tree parameters were continuously modified until the accuracy requirements were met, at which point the results were exported as vector files.

**TABLE 2:** Feature parameters for surface type classification

Features	Feature characteristics	Major purpose
Spectral information	Mean brightness	To classify alpine shrub and alpine meadow, bare soil, and pika burrow
	Excess green (EXG) Normalized green‐red difference index (NGRDI) Red green‐blue vegetation index (RGBVI)	To classify vegetation and nonvegetation
Geometry information	Shape index	To classify bare soil and rodent burrow
Texture information	GLCM contrast	To classify alpine shrub and alpine meadow

Four hundred and eighty ground truth samples of different landscape types (200 alpine meadow, 160 alpine shrub, 80 bare soil, and 40 plateau pika burrow) randomly generated by GIS software were selected and identified in the 45 quadrats to evaluate the classification accuracy. The results show that the average overall accuracy was 93.59%, and the kappa coefficient was 0.91; these values satisfied the research requirements.

#### 2.2.2 Selection and calculation of landscape pattern metrics

We used the characteristics of the pika burrows and other surface types to select six landscape pattern metrics (LPMs) at the class level, representing attributes of the landscape such as area, fragmentation, stability, and connectivity (Table 3). We used the R package *landscapemetrics* (Hesselbarth et al., 2019) to calculate the LPMs for each quadrat.

**TABLE 3:** Selected landscape pattern metrics, and the calculation and interpretation of these metrics

Metric/acronym	Calculation/unit/description	Indication
ED (Edge Density)	Calculation: the edge density equals the sum of all edges of class *i* in relation to the landscape area Unit: m/m^2^ Description: equals ED = 0 if only one patch is present (and the landscape boundary is not included) and increases, without limit, as the landscapes become more patchy	Fragmentation Stability
ENN_MN (mean of Euclidean nearest‐neighbor distance)	Calculation: ENN equals the distance (m) to the nearest neighboring patch of the same type, based on shortest edge‐to‐edge distance Unit: m Description: approaches ENN_MN = 0 as the distance to the nearest neighbor decreases; that is, patches of the same class *i* are more aggregated. Increases, without limit, as the distance between neighboring patches of the same class *i* increases; that is, patches are more isolated	Connectivity Configuration
LPI (largest patch index)	Calculation: It is the percentage of the landscape covered by the corresponding largest patch of each class *i* Unit: % Description: Largest patch index at the class level quantifies the percentage of total landscape area comprised by the largest patch. As such, it is a simple measure of dominance	Dominance
PD (patch density)	Calculation: PD equals the number of patches of the corresponding patch type divided by total landscape area Unit: Number/m^2^ Description: increases as the landscape gets more patchy. Reaches its maximum if every cell is a different patch	Fragmentation
AREA_MN (mean of patch area)	Calculation: The metric summarizes each class as the mean of all patch areas belonging to class *i* Unit: m^2^ Description: approaches AREA_MN = 0 if all patches are small. Increases, without limit, as the patch areas increase	Composition Patch structure
NP (number of patches)	Calculation: number of patches in the landscape of patch type Unit: None Description: NP equals the number of patches of the corresponding patch type	Fragmentation

### 2.3 Statistical analysis

The characteristics of landscapes at different stages of degradation are presented as the average ± standard deviation. One‐way analysis of variance (ANOVA) and a multicomparison of the least significant difference (LSD) test were performed using the SPSS 17.0 statistical software package (SPSS Inc.) to determine differences at a *p* = .05 level. Spearman's rank correlation was used to study the relationship between the pattern of pika burrows and other surface types.

## 3 RESULTS

### 3.1 Changes in area and spatial pattern of different surface types along the degradation sequence

Alpine shrub meadows have similar area structures in GE and LD. Both alpine meadows and alpine shrubs are absolutely dominant landscape types. The area of bare soil only accounts for 0.32–0.71%, while pika burrows do not appear. At MD level, the proportion of alpine shrub decreased significantly by 85% (*p* < .05), and the area of alpine meadow increased significantly by 66% (*p* < .05) and became the dominant landscape type. At HD level, alpine shrub has degraded to the point where there is no trace of its existence on the surface and some of the alpine meadow was degraded to bare soil, resulting in an almost 84 times increase in the area of bare soil, and the pika burrow area began to increase. At SD, the surface landscape consisted of alpine meadows, bare soil, and pika burrows. The area of alpine meadows declined further, while the area of bare soil increased by 37% compared with the HD stage and the area of pika burrows increased threefold (Table 4).

**TABLE 4:** Changes in area proportion of surface types at different levels of degradation (%)

Degradation level	Alpine shrub	Alpine meadow	Bare soil	Pika burrow
GE	43.20 ± 12.26^a^	56.09 ± 11.86^c^	0.71 ± 0.52^b^	–
LD	43.99 ± 17.15^a^	55.74 ± 16.84^c^	0.32 ± 0.74^b^	–
MD	6.32 ± 3.56^b^	93.38 ± 3.67^a^	0.34 ± 0.35^b^	0.00 ± 0.00^b^
HD	–	71.32 ± 7.22^b^	28.58 ± 7.21^a^	0.10 ± 0.08^b^
SD	–	60.60 ± 11.62^bc^	39.12 ± 11.64^a^	0.31 ± 0.11^a^

Abbreviations: GE, grazing exclusion; HD, heavy degradation; LD, light degradation; MD, moderate degradation; SD, severe degradation.

Lower case letters represent significant differences (*P* < .05) in area proportion for the same surface type at different levels of degradation.

The NP and PD of alpine meadows can represent the degree of landscape fragmentation; they both showed little change in LD and a significant decrease in MD (*p* < .05), when the alpine meadow occupied the habitat of the alpine shrub and became the dominant landscape type. During the HD stage, the NP and PD of the alpine meadows increased significantly and sharply (*p* < .05), which corresponded to the degradation of the alpine meadow and the expansion of the bare soil. During the SD stage, NP and PD decreased again, indicating the continuous loss of isolated meadow patches due to the connectivity and expansion of bare soil patches. Area_MN of the meadow patches changed little in LD and increased rapidly in MD, which in turn decreased rapidly in HD and increased slightly in the SD stage (Table 5). This further illustrates the fact that alpine meadows tend to fragment as they degrade.

**TABLE 5:** Spatial pattern changes of surface types in different degradation levels

	NP (number)	PD (number/m^2^)	Area_MN (m^2^)	LPI (%)	ED (m/m^2^)
Alpine meadow					
GE	292.11 ± 198.97^c^	2.82 ± 1.74^b^	0.33 ± 0.37^a^	48.22 ± 17.87^b^	9.67 ± 2.73^b^
LD	233.13 ± 132.43^c^	2.52 ± 1.46^b^	0.34 ± 0.26^a^	47.21 ± 25.45^b^	8.45 ± 1.14^b^
MD	70.90 ± 54.63^c^	0.55 ± 0.44^c^	3.94 ± 4.75^a^	93.25 ± 3.77^a^	4.16 ± 2.03^c^
HD	1838.80 ± 819.04^a^	17.21 ± 7.70^a^	0.06 ± 0.05^a^	66.37 ± 13.67^b^	13.59 ± 2.67^a^
SD	1171.73 ± 1080.40^b^	10.35 ± 9.76^ab^	0.17 ± 0.23^a^	46.24 ± 21.13^b^	12.08 ± 5.07^ab^
Alpine shrub					
GE	294.11 ± 76.01^a^	2.73 ± 0.79^b^	0.16 ± 0.06^a^	24.34 ± 15.37^a^	9.38 ± 3.02^a^
LD	427.38 ± 128.92^a^	4.40 ± 1.38^a^	0.12 ± 0.07^a^	18.17 ± 18.44^a^	8.35 ± 1.22^ab^
MD	453.70 ± 225.52^a^	4.25 ± 2.26^ab^	0.02 ± 0.01^b^	0.36 ± 0.22^b^	4.04 ± 2.05^a^
Bare soil					
GE	28.00 ± 26.17^c^	0.26 ± 0.26^b^	0.03 ± 0.02^a^	0.14 ± 0.13^b^	0.30 ± 0.24^b^
LD	6.88 ± 7.22^c^	0.08 ± 0.08^b^	0.02 ± 0.03^ab^	0.06 ± 0.12^b^	0.09 ± 0.18^b^
MD	48.89 ± 61.25^c^	0.47 ± 0.63^b^	0.01 ± 0.01^b^	0.11 ± 0.17^b^	0.21 ± 0.19^b^
HD	1396.30 ± 529.38^a^	13.76 ± 5.22^a^	0.02 ± 0.01^a^	7.14 ± 6.37^a^	13.51 ± 2.65^a^
SD	803.45 ± 592.36^b^	7.51 ± 5.95^a^	0.11 ± 0.09^a^	19.35 ± 12.92^a^	12.27 ± 5.11^a^

Abbreviations: GE, grazing exclusion; HD, heavy degradation; LD, light degradation; MD, moderate degradation; SD, severe degradation.

Lower case letters represent significant differences (*P* < .05) in landscape patterns for the same surface type at different levels of degradation.

LPI indicates the proportion of the area of the largest patch in the landscape, which can reflect the stability and resilience potential of a certain landscape type. LPI in alpine meadows did not change much in LD, but increased significantly in MD (*p* < .05), indicating that the stability of the meadow landscape increases significantly at this stage, while it decreased continuously in HD and SD, indicating that the stability and resilience potential of alpine meadows continue to decline at these stages (Table 5). The ED reflects the extent to which the landscape is disturbed by the outside world, with the ED of alpine meadows decreased continuously in LD and MD and increased significantly in HD (*p* < .05), followed by a slight decrease in SD, indicating that alpine meadows are most strongly disturbed by other types in the HD stage (Table 5).

NP in alpine shrub continued to increase in LD and MD, while PD also increased significantly in LD (*p* < .05) and decreased slightly in MD. Area_MN and LPI continued to decrease and reached a minimum in MD. This indicates that the intact shrub is constantly being broken up into small isolated patches. The ED of the alpine shrub also showed a continuous decline, indicating that its boundaries tend to be simpler (Table 5).

Bare soil is one of the main indicators of alpine grassland degradation, and its landscape pattern generally changes significantly during the HD stage. The NP and PD of bare soil underwent a decrease followed by an increase at both the LD and MD stages, but only increased significantly and dramatically (*p* < .05) at HD, before decreasing slightly at SD. Area_MN, on the contrary, increased significantly in SD, and LPI continued to increase in HD and SD, indicating that bare soil expanded rapidly in both stages, occupying the original habitat of the alpine meadow. The ED of bare soil increased significantly in HD (*p* < .05) and then decreased slightly in SD, indicating that the bare soil boundary was complex and unstable under this stage and that degradation may still be developing (Table 5).

### 3.2 Changes in the spatial pattern of plateau pika burrow along the degradation gradient

**FIGURE 4:**
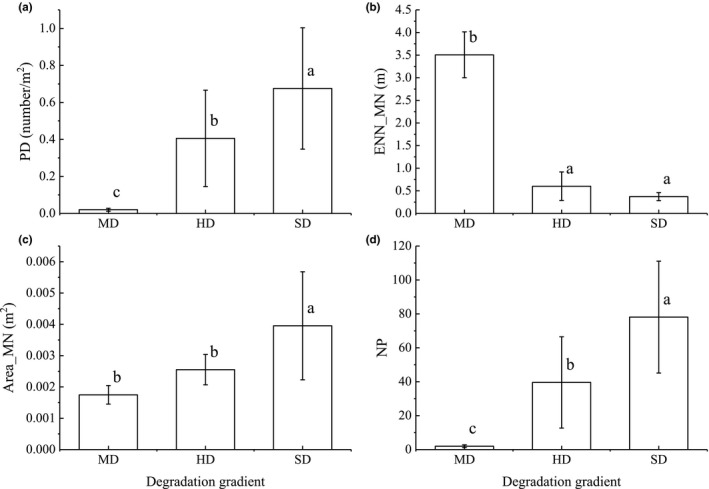
Changes in the spatial pattern of pika burrow between different degradation levels in alpine grassland. Abbreviations: Area_MN, mean of patch area; ENN_MN, mean of Euclidean nearest‐neighbor distance; HD, heavy degradation; MD, moderate degradation; NP, number of patches; PD, patch density; SD, severe degradation

The pika burrows did not occur at the land surface at the GE and LD stages, but as grassland degradation increased, they gradually spread from being isolated and occasional occurrences to being distributed across the surface landscape. The number and density of pika burrows continuously and significantly increased from MD (2 ± 0.82 and 0.02 ± 0.01 number/m^2^) to HD (39.6 ± 26.93 and 0.41 ± 0.26 number/m^2^), and increased further at SD (78.10 ± 32.98 and 0.68 ± 0.33 number/m^2^). The patch size (Area_MN) was the largest (0.004 ± 0.0017 m^2^) at SD. The linear distance (ENN_MN) between burrows shortened rapidly at the HD compared with MD level, from 3.51 ± 0.51 m to 0.60 ± 0.31 m. The distance between burrows reduced slightly further between the HD and SD (0.37 ± 0.09 m), but not significantly (Figure 4).

### 3.3 The relationship between the spatial pattern of plateau pika burrow and other land surface types

**FIGURE 5:**
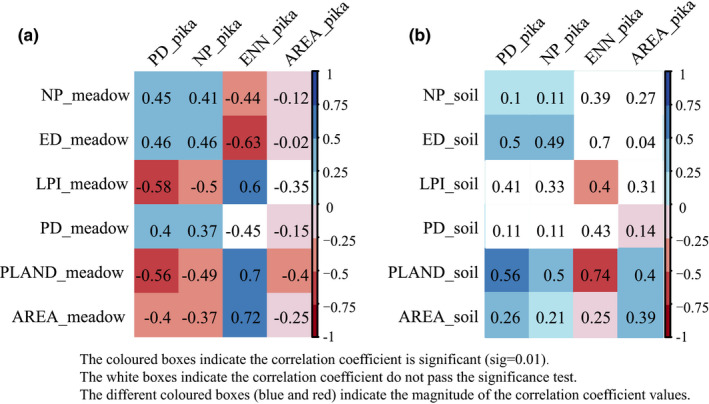
Correlations between the spatial pattern of pika burrow and (a) alpine meadow, and (b) bare soil (sig = 0.01). Note: Colored boxes represent significant correlations (*p *< .01)

There is a significant correlation between the spatial pattern of alpine meadow and pika burrows, except for Area_MN. LPI and PLAND for alpine meadow were more closely related to the spatial pattern of pika burrow, with LPI having a significant negative correlation with PD for pika burrows (Figure 5), and a significant positive correlation with ENN for pika burrows (Figure 5). PLAND for alpine meadow had similar characteristics, but was more closely related to ENN for pika burrow, while its correlation coefficient with PD for pika burrows was less than that for LPI. Both ED and AREA_MN for alpine meadow were significantly negatively and significantly positively correlated with ENN_MN for pika burrows. The decrease in the proportional area of alpine meadow, and the shrinkage of its dominant patches, may have led to an increase in the number and density of pika burrow, as well as a decrease in their connectivity (Figure 5a).

The relationship between most spatial pattern features of bare soil and pika burrows is less clear than for alpine meadows and pika burrows. Only PLAND and AREA_MN correlate with all pattern indices for pika burrows at significant levels. There were a significant negative correlation between PLAND for bare soil and ENN_MN for pika burrows, and a significant positive correlation with other indices. The ED of bare soil was significantly and positively correlated with the PD and NP for pika burrows (Figure 5b).

As the consistently dominant landscape type, alpine meadow had a significantly greater effect on the spatial pattern of pika burrows than bare soil did. The decrease in PLAND and LPI for alpine meadow caused an increase in the number and density of pika burrows, while the increase in PLAND for bare soil caused the same effect.

**FIGURE 6:**
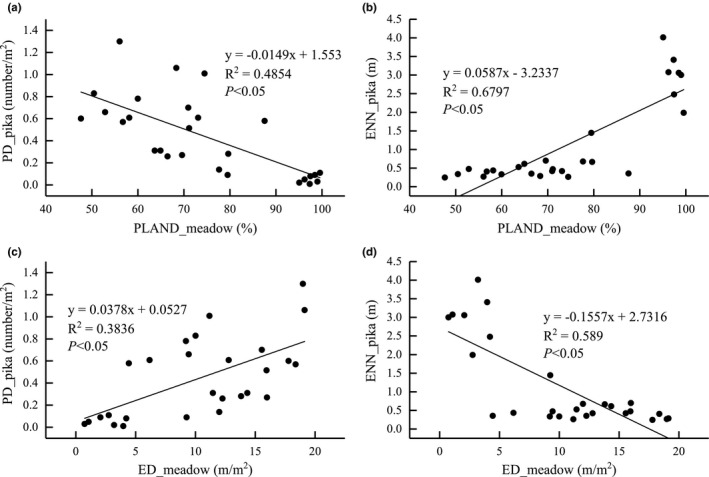
Relationship between PLAND and ED for alpine meadow and PD and ENN for pika burrows. Abbreviations: ED, edge density; ENN_MN, mean of Euclidean nearest‐neighbor distance; PD, patch density; PLAND, percentage of landscape

We used PLAND and ED, which are closely related to the spatial pattern of pika burrows, to represent the area and edge characteristics of alpine meadow, respectively, and used PD and ENN, which represent the density and connectivity of pika burrows, to quantitatively analyze the relationship between spatial pattern of alpine meadow and pika burrows. There was a significant linear relationship between PLAND for alpine meadow and PD and ENN for pika burrow. For every 10% decrease in PLAND of alpine meadow, the PD of pika burrows increased by 0.15 number/m^2^, and ENN for pika burrows increased by 0.59 m. There was also a significant linear relationship between ED for alpine meadow and PD and ENN for pika burrows. For every 10 m/m^2^ increase in ED for alpine meadow, PD for pika burrows increased by 0.38 number/m^2^, while ENN shrank by 1.56 m (Figure 6).

## 4 DISCUSSION

The two‐layered structure of shrub and meadow of a landscape in pristine condition degraded to a landscape pattern dominated by meadows and bare soil, with pika burrows throughout as the level of landscape degradation increased. Moderate degradation was the key stage of change in the surface landscape pattern of alpine shrub meadows, as the area of shrub declined sharply, meadow became the dominant landscape type, and pika burrows began to appear. As degradation increased, meadows tended to fragment, while bare soil expanded and encroached on the meadow landscape, and pika burrows became a pervasive landscape feature.

The density, average size, and number of pika burrows increased significantly with the degradation of alpine shrub meadow, while the linear distance between burrows decreased significantly. Changes in the size of pika burrows, and the distance between them are related to the migration, development, and reproduction of pika populations. As pika migrate, the number of new burrows inevitably increases, and as pikas become larger and increase in number due to constant breeding, their burrow area increases to accommodate their size and the growing number of family members. The increasing number of burrows in a limited habitat inevitably leads to a reduction in the distance between burrows (Cao et al., 2019; Qu et al., 2018).

The results of this study showed a density of 5500 pika burrows/ha in degraded alpine shrub meadow, whereas surveys conducted in the western part of the southern Qilian Mountains have shown that the average number of pika burrows in normal grassland is 300–800 burrows/ha (Qin, Sun, et al., 2020; Qin et al., 2019; Yi et al., 2016). In contrast, studies of degraded alpine grassland in the hinterland of QTP found the average number of pika burrows to be 2700–4775 burrows/ha (Liu et al., 2017; Tian et al., 2019; Wang et al., 2020; Wei et al., 2013), and in the degraded grasslands of the eastern QTP, the density of pika burrows can even reach 7066 burrows ha^−1^ (Zhang et al., 2021), which is close to our results. The density of pika burrows has been proven to be significantly correlated with the coverage of vegetation and bare soil (Tang et al., 2019; Zhang et al., 2021), and it is also a valid indicator to estimate the population size of pika in the plateau. There is evidence that increases in the density of plateau pika populations lead to increased reductions in grassland aboveground biomass, soil carbon, and nitrogen (Liu et al., 2017; Qin, Yi, et al., 2020; Yi et al., 2016). This suggests that the increase in the population of plateau pika does have a negative impact on grassland ecosystems, but that grassland degradation is still a complex process involving multiple factors, and that pika population may only exacerbate grassland degradation when a certain threshold is exceeded.

We found that the average area of a pika burrow opening was 0.003 m^2^, which is smaller compared with other studies (Ma et al., 2011; Pech et al., 2007). We also found that the average linear distance between pika burrows across all the stages of degradation was 0.49 m. However, there are few examples of studies for comparison, due to the limited means of investigation. Tang et al. (2019) investigated the spatial connectivity of pika burrows using the COHESION index, but as this index is a relative measure with an upper limit of 100, it is difficult to compare it with our study.

In this paper, we found that the landscape pattern of alpine meadows was closely related to that of pika burrows. Higher proportions of meadow area, large meadow patches, and larger average meadow patch size were all able to inhibit the development of pika burrows. Tang et al. (2019) also found that a similar relationship exists between vegetation cover and the number of pika holes. This is because dense vegetation is not conducive to pika escaping or avoiding predators, and they therefore prefer habitats that provide an open view and lower grass height, such as degraded grassland (Liu et al., 2003). Therefore, it is now generally accepted that pika does not cause grassland degradation. However, the activities of pikas do accelerate the rate of degradation in alpine grassland by reducing productivity and altering the structure of plant communities through their feeding and clipping effects. Furthermore, their burrow excavations of burrows can directly occupy grassland growth space and therefore, together with the alteration of soil properties, lead to an increase in the area of bare soil and a decrease in the proportion of grassland area (Tang et al., 2019; Yu et al., 2017).

A further linear relationship was constructed to show the key threshold for changes in pika burrow patterns; that is, for every 10% increase in the proportion of meadow area, the density of pika burrows decreases by 0.15 burrows/m^2^, while the distance between burrows increases by 0.59 m. Based on the average density of pika burrows in the heavily degraded stage of this study, it can be inferred that the critical limit for the proportion of alpine meadow area is approximately 77%, and if it falls below this value, then plateau pika activity may become difficult to control, which is closer to the results of Tang et al. (2019) (60% grass cover as the critical threshold).

## 5 CONCLUSION

Using high‐resolution UAV images, this study provides a quantitative assessment of changes in the spatial pattern of plateau pika burrows along the alpine shrub meadow degradation gradient. The degradation of the alpine shrub meadow leads to a dramatic change in the surface landscape pattern, with the two‐layered structure of shrub and meadow changing to a landscape pattern where meadow and bare soil coexist and pika burrows are scattered throughout. Moderate degradation is the starting point for the dramatic changes in pika burrows and the loss of shrub. The increasing number and size of pika burrows naturally lead to an increase in their density and a reduction in their distance. Maintaining a certain proportion of meadow area is a key measure to prevent uncontrolled pika burrow increase. Therefore, improving grassland management is the key to preventing grassland degradation and pika activity from threatening the sustainable use of alpine grassland. UAV technology can provide a new observation tool for grassland degradation studies on the Qinghai–Tibet Plateau, and has great advantages and potential for plateau pika surveys.

## CONFLICT OF INTEREST

No conflict of interest exists in the submission of this manuscript, and the manuscript is approved by all authors for publication.

## AUTHOR CONTRIBUTION

**Dawen Qian:** Conceptualization (lead); Data curation (lead); Formal analysis (lead); Funding acquisition (lead); Investigation (lead); Methodology (lead); Software (lead); Visualization (equal); Writing‐original draft (lead); Writing‐review & editing (equal). **Qian Li:** Methodology (equal); Resources (equal); Supervision (equal); Writing‐review & editing (equal). **Bo Fan:** Resources (equal); Software (equal); Validation (equal); Writing‐review & editing (supporting). **Yuting Lan:** Investigation (equal); Resources (equal); Validation (equal); Writing‐review & editing (equal). **Guangmin Cao:** Conceptualization (equal); Funding acquisition (equal); Methodology (equal); Project administration (equal); Supervision (equal).

## Data Availability

Data are available on Dryad Digital Repository (https://doi.org/10.5061/dryad.vx0k6djsd).
